# Revisiting Functional Connectivity for Infraslow Scale-Free Brain Dynamics Using Complex Wavelets

**DOI:** 10.3389/fphys.2020.578537

**Published:** 2021-01-07

**Authors:** Daria La Rocca, Herwig Wendt, Virginie van Wassenhove, Philippe Ciuciu, Patrice Abry

**Affiliations:** ^1^CEA, NeuroSpin, University of Paris-Saclay, Paris, France; ^2^Inria Saclay Île-de-France, Parietal, University of Paris-Saclay, Paris, France; ^3^IRIT, CNRS, University of Toulouse, Toulouse, France; ^4^INSERM U992, Collège de France, University of Paris-Saclay, Paris, France; ^5^Univ. Lyon, ENS de Lyon, Univ. Claude Bernard, CNRS, Laboratoire de Physique, Lyon, France

**Keywords:** human brain temporal dynamics, functional connectivity, infraslow, arrhythmic, scale-free, fractal connectivity, complex-wavelet, MEG data

## Abstract

The analysis of human brain functional networks is achieved by computing functional connectivity indices reflecting phase coupling and interactions between remote brain regions. In magneto- and electroencephalography, the most frequently used functional connectivity indices are constructed based on Fourier-based cross-spectral estimation applied to specific fast and band-limited oscillatory regimes. Recently, infraslow arrhythmic fluctuations (below the 1 Hz) were recognized as playing a leading role in spontaneous brain activity. The present work aims to propose to assess functional connectivity from fractal dynamics, thus extending the assessment of functional connectivity to the infraslow arrhythmic or scale-free temporal dynamics of M/EEG-quantified brain activity. Instead of being based on Fourier analysis, new Imaginary Coherence and weighted Phase Lag indices are constructed from complex-wavelet representations. Their performances are first assessed on synthetic data by means of Monte-Carlo simulations, and they are then compared favorably against the classical Fourier-based indices. These new assessments of functional connectivity indices are also applied to MEG data collected on 36 individuals both at rest and during the learning of a visual motion discrimination task. They demonstrate a higher statistical sensitivity, compared to their Fourier counterparts, in capturing significant and relevant functional interactions in the infraslow regime and modulations from rest to task. Notably, the consistent overall increase in functional connectivity assessed from fractal dynamics from rest to task correlated with a change in temporal dynamics as well as with improved performance in task completion, which suggests that the complex-wavelet weighted Phase Lag index is the sole index is able to capture brain plasticity in the infraslow scale-free regime.

## 1. Introduction

### 1.1. Human Brain Univariate Temporal Dynamics

The dynamics of Human brain activity can be studied non-invasively using electro- and magnetoencephalography (EEG and MEG, respectively). Interpreted as resulting from the synchronous activation of neuronal populations in specific frequency bands, these fluctuations are often analyzed as fast (10 Hz and above) oscillatory rhythms now associated with cognitive functions, such as perception, attention, or decision making (cf. e.g., Freeman, 2000; Jensen and Colgin, 2007), described by band-limited models, and analyzed by classical Fourier transform-based spectral analysis.

At the turn of the 21st century, the large-band infraslow activity of the brain (typically below 1 Hz), which for long had been considered as either instrumental or head-movement noise, received growing interest; it has now been documented as a prominent part of recorded electromagnetic brain signals and a critical component of brain activity (Gong et al., 2003; Stam and De Bruin, 2004; Vanhatalo et al., 2004; Miller et al., 2009; Werner, 2010). This large-band infraslow activity in the brain differs significantly from band-limited oscillations in the sense that it is not characterized by specific frequencies or scales of times but rather corresponds to arrhythmic, or scale-free, temporal dynamics. While exact scale-free dynamics remains debatable (Dehghani et al., 2010; Ignaccolo et al., 2010), it has been proposed by an abundant literature (cf. e.g., Vanhatalo et al., 2004; Dehghani et al., 2010; He et al., 2010; Van de Ville et al., 2010; He, 2011, 2014; Zilber et al., 2012; Buzsáki and Mizuseki, 2014; Gadhoumi et al., 2015; La Rocca et al., 2018b) that infraslow macroscopic brain activity is better described by a scaling exponent (historically the power-law exponent of the Fourier spectrum and more recently and relevantly the selfsimilarity exponent *H*) that relates together dynamics across a large continuum of slow time scales (or low frequencies). While most oscillatory regimes are only observed in evoked activity, elicited by stimuli, infraslow scale-free brain temporal dynamics are persistent, observed both at rest and during task performance or even in unconscious states (e.g., sleep stages). It was also shown that infraslow scale-free brain temporal dynamics are modulated when contrasting rest and task-related brain activity, task-inducing systematically a decrease in *H* and *faster* infraslow dynamics (Bhattacharya and Petsche, 2001; Linkenkaer-Hansen et al., 2004; Vanhatalo et al., 2004; Popivanov et al., 2006; Bianco et al., 2007; Buiatti et al., 2007; He et al., 2010; Zilber et al., 2013; La Rocca et al., 2018b). Infraslow scale-free brain activity has thus been hypothesized to be functionally associated with neural excitability (He, 2014). Altered scale-free brain dynamics has also been reported in a specific condition, such as Alzheimer's disease for which larger selfsimilarity exponents were reported in multiple brain areas (e.g., lateral temporal lobes, insula, etc.) involved early in the neurodegenerative process (Maxim et al., 2005).

Infraslow arrhythmic brain activity can be efficiently described with large-band scale-free models, such as selfsimilar processes (fractional Brownian motion and fractional Gaussian noise) (Mandelbrot and van Ness, 1968). It is also now well-established and documented that, while Fourier analysis can be used to assess 1/*f* power-law spectra at low frequencies, accurate and robust assessments of scale-free dynamics requires replacing Fourier-based spectral estimation with multiscale wavelet analysis. Interested readers are referred to Flandrin (1992), Muzy et al. (1993), Veitch and Abry (1999), Kantelhardt (2008), and Abry et al. (2019b) for methodological developments and to Ciuciu et al. (2008, 2012, 2014), and La Rocca et al. (2018b) for applications to neuroimaging data. Further, it has recently been shown that the self-similar description of scale-free temporal dynamics could be enriched by combining the concept of multifractality with that of selfsimilarity (Wendt et al., 2007; Abry et al., 2019b), requiring the use of wavelet-leaders, consisting of non-linear non-local transforms of wavelet coefficients, for practical analysis. The potential interest of multifractality for the analysis of fMRI and M/EEG signals has been investigated in e.g., Shimizu et al. (2004), Popivanov et al. (2005), Popivanov et al. (2006), Shimizu et al. (2007), Ciuciu et al. (2008, 2012), Proekt et al. (2012), and La Rocca et al. (2018b).

### 1.2. Human Brain Multivariate Temporal Dynamics: Functional Connectivity

Remote brain regions are known to interact within large scale functional networks (e.g., the default Mode Network at rest), which mediate the information flow inside the brain integrating the activity of functionally segregated modules that are activated in particular mental states, task execution, or health condition (Power et al., 2011). These interactions (correlations, delays, phase synchronization, etc.) between different brain regions are quantified by indices of similarity computed from signals collected in each region and are referred to as *functional connectivity*. Assessing functional connectivity thus entails performing a multivariate analysis of the temporal recordings, thus complementing univariate analysis of each signal separately. Classically, functional connectivity is assessed mostly from band-limited signals reflecting the oscillatory activity of the brain, by measures of cross (bivariate) second-order statistics (correlation coefficient, cross-correlation function, etc.). However, M/EEG measurements suffer from the so-called *volume conduction effects*: Linearity in Maxwell equations and electromagnetic quasi-static approximation (for the forward model below 100 Hz) induces a linear mixing of electromagnetic sources on M/EEG sensors with negligible temporal delays. Close-by EEG electrodes or SQUID MEG sensors thus redundantly capture brain activity from a given current cortical dipole, inducing spurious correlations amongst recordings and thus precluding a relevant assessment of functional connectivity (Nolte et al., 2004; Stam et al., 2007; Vinck et al., 2011). Source-space reconstructed signals are documented to still suffer from residual volume conduction effects because of the approximate and imperfect nature of inverse problem resolutions (Siebenhühner et al., 2016; Palva et al., 2018). The design of indices robust to such spurious correlations has been based on measuring average phase delays, such as in the Phase Locking Value (Stam et al., 2007), and also naturally calls for the use of Fourier-based cross-spectral estimation. Indeed, the Fourier transform, being by definition based on complex numbers, permits us to automatically incorporate phases and thus delays in the assessment of functional connectivity: zero delay between correlated signals corresponds to zero phase and imaginary part but non-zero real part for the cross-Fourier spectrum (on average). Therefore, the moduli of the cross-Fourier spectrum and the coherence function (F-COH) are affected by volume conduction effects, but their imaginary parts and phases are robust to such spurious effects and in theory depart from zero only for dependent sources with actual delays: a crucial property for assessing functional connectivity. This observation has led to the design, study, and use of the Imaginary Coherence function (F-ICOH) (Nolte et al., 2004) and the (weighted-)Phase Lag Index (F-wPLI) (Vinck et al., 2011) as relevant indices to assess functional connectivity for the band-limited oscillatory brain activity measured by M/EEG measurements. Interested readers are referred to e.g., Engel et al. (2001), Varela et al. (2001), Nolte et al. (2004), Stam et al. (2007), Vinck et al. (2011), and Siegel et al. (2012) for thorough reviews and further details (see also section 2.1 for definitions). Beyond second-order statistics and linear correlation, higher-order (non-linear) dependencies have also been investigated using directed partial correlations; moreover, the Granger causality approach has been used to infer causal links, see Sakkalis (2011) for a review.

Functional connectivity has so far mainly been measured via the band-limited oscillatory activity of the brain and has hardly been applied to characterize the infraslow arrhythmic brain activity. Preliminary attempts in that direction (Achard et al., 2008; Ciuciu et al., 2014), though based on wavelet representation, remained tied to the coherence function, hence essentially to direct correlation, and are thus severely impaired by volume conduction effect in functional connectivity assessment in M/EEG. This lack of functional connectivity tools dedicated to the infraslow regime is partly due to the role infraslow arrhythmic temporal dynamics to brain activity remaining controversial but also because conceptual and practical tools reconciling the modeling and analysis of both multivariate and scale-free dynamics were lacking. This situation changed recently with the theoretical definition and formal study of multivariate selfsimilarity (Didier and Pipiras, 2011) as well as with the design and assessment of multivariate wavelet transform based practical tools (Wendt et al., 2017; Abry and Didier, 2018a,b; Abry et al., 2019a,b), thus permitting the investigation of functional connectivity within the infraslow arrhythmic brain activity, at the core of the present work.

### 1.3. Goals, Contributions, and Outline

The present work aims to revisit the analysis of functional connectivity in human brain activity in two ways:

First, functional connectivity assessment will be based on the on-going (or spontaneous) infraslow arrhythmic (or scale-free) activity of the human brain rather than on stimulus-induced band-limited oscillatory faster rhythms. This will be referred to as *functional connectivity assessed from fractal dynamics* (see La Rocca et al., 2018a for a preliminary attempt).

Second, indices quantifying functional connectivity from fractal dynamics will be constructed from multivariate complex wavelet transforms rather than from Fourier-based cross-spectral analysis. The key intuitions underlying the design of these indices are double: Based on wavelet transforms, these tools will inherit from their well-documented performance and robustness for the analysis of scale-free dynamics (Flandrin, 1992; Abry and Veitch, 1998; Veitch and Abry, 1999, 2001; Abry et al., 2000, 2019b); Complex wavelets allow us to incorporate phase information in the analysis of multivariate cross-temporal dynamics.

To that end, after a brief recall of Fourier-based spectral estimation and the classical Fourier-based functional connectivity indices (F-ICOH and F-wPLI) in section 2.1, Complex wavelet transforms and the corresponding Complex Wavelet-based functional connectivity indices (W-ICOH and W-wPLI) are defined in section 2.2. The performance of several Complex Wavelet-based functional connectivity indices proposed here are compared against the others, and against their corresponding Fourier counterparts, by means of Monte Carlo numerical simulations, involving a large number of independent drawings of synthetic signals, sampled from stochastic processes commonly used to model scale-free temporal dynamics, multivariate fractional Brownian motions, and multivariate fractional Gaussian noises (cf. section 2.3). Several scenarios (different temporal dynamics, connectivity networks, additive trends) are investigated to assess the interest and relevance of the proposed Complex Wavelet indices (W-ICOH and W-wPLI) compared to Fourier-based ones in terms of estimation performance and robustness to trends.

The proposed Complex Wavelet indices assessing functional connectivity from fractal dynamics are extensively tested on MEG data, collected on 36 individuals, both at rest and during a visual discrimination learning task. The experimental data are described in section 3 (see also Zilber et al., 2014).

Analyses of functional connectivity assessed from fractal dynamics within infraslow arrhythmic cross temporal dynamics regime, ranging from 0.1 to 1.5 Hz for this data set (La Rocca et al., 2018b), are reported in section 4 and discussed in section 5. The proposed Complex Wavelet indices are demonstrated to have a high sensitivity in capturing significant and meaningful group-level functional connectivity assessed from fractal dynamics networks both at rest and during task performance, which present long-range spatial interactions between fronto-occipital and temporo-parietal brain regions. Further, a significant increase in functional connectivity assessed from fractal dynamics is shown to be positively correlated with behavioral performance in the task and to be reinforced by the training stage and thus by learning. Finally, our results suggest an interplay between temporal and spatial dynamics: Arrhythmic infraslow brain activity evolves from strongly and globally structured slow temporal dynamics for each region individually at rest, related across the brain by a clear functional network, to faster and less globally structured temporal dynamics per region, yet with significantly stronger spatial couplings across the brain, during a task.

The proposed Complex Wavelet tools constitute, to the best of our knowledge, the first operational tools for a relevant assessment of functional connectivity from fractal dynamics, i.e., functional connectivity in scale-free cross-temporal dynamics for the large-band infraslow arrhythmic brain activity recorded in M/EEG. Matlab codes, designed and implemented by ourselves, for the synthesis of multivariate scale-free synthetic data and for the computation of Complex Wavelet-based indices to assess functional connectivity from fractal dynamics, will be made publicly available at the time of publication.

## 2. Methodology: Functional Connectivity Assessment

### 2.1. Frequency Domain Functional Connectivity Assessment

The *M*-variate data (*X*_*m*_(*t*)_*m* = 1, ..., *M*_, *t* ∈ ℝ) available for analysis are assumed to be real-valued finite power realizations of stochastic processes with well-defined power cross-spectral density $S_{m,m^{\prime }}\left(f\right)$. *The Welch periodogram* constitutes one of the classical non-parametric spectral estimation procedures (Papoulis, 1977), and it is based on the use of a windowed Fourier transform. This Fourier-based estimate *S*^(*F*)^ of the cross-spectrum *S* is indeed defined as a time average of the squared-moduli of the windowed (or short-time) Fourier transform coefficients *g*_*X*_(ℓ, *k*) = ∫*X*(*t*)ϕ_ℓ, *k*_(*t*)*dt*:

(1) $$\begin{array}{l} S_{m,m^{\prime }}^{\left(F\right)}\left(f=\ell \nu_{0}\right)=\underset{k}{\sum }g_{X_{m}}\left(\ell ,k\right)g_{X_{m^{\prime }}}^{*}\left(\ell ,k\right), \end{array}$$

where ϕ_ℓ,*k*_(*t*) = ϕ(*t* − *kT*_0_) exp (−2*1ℓν*_0_*t*) denotes the collection of translated and frequency-shifted templates of a reference pattern ϕ(*t*), and *T*_0_ and ν_0_ are positive constants that can be arbitrarily chosen provided that they satisfy *T*_0_ ν _0_ ≤ 1/(4π).

Straightforward calculations yield

(2) $$\begin{array}{l} 𝔼S_{m,m^{\prime }}^{\left(F\right)}\left(\ell \nu_{0}\right)=\int S_{m,m^{\prime }}\left(\ell \nu_{0}-f\right)|\tilde{\phi }\left(f\right){|}^{2}df, \end{array}$$

with $\tilde{\phi }$ denoting the Fourier transform of ϕ and 𝔼 the ensemble average. This thus shows that $S_{m,m^{\prime }}^{\left(F\right)}$ provides a biased estimate of $S_{m,m^{\prime }}\left(f\right)$. The time and frequency resolutions of the functions ϕ_ℓ,*k*_ being uniformly controlled by the choice of the function ϕ, *S*^(*F*)^ achieves a fixed *absolute-frequency resolution* multivariate spectral analysis.

From $S_{m,m^{\prime }}^{\left(F\right)}\left(f\right)$, three functions are classically involved in functional connectivity assessment, the modulus (F-COH), the Imaginary (F-ICOH) part of the coherence function (Nolte et al., 2004), and the weighted Phase Lag Index (F-wPLI) (Vinck et al., 2011) (with ℑ the imaginary part of a complex number):

(3) $$\begin{array}{l} {\text{F-COH}}_{m,m^{\prime }}\left(f\right)≜\frac{S_{m,m^{\prime }}^{F}\left(f\right)}{\sqrt{S_{m,m}^{F}\left(f\right)S_{m^{\prime },m^{\prime }}^{F}\left(f\right)}}, \end{array}$$

(4) $$\begin{array}{l} {\text{F-ICOH}}_{m,m^{\prime }}\left(f\right)≜\frac{ℑ\left\{S_{m,m^{\prime }}^{F}\left(f\right)\right\}}{\sqrt{S_{m,m}^{F}\left(f\right)S_{m^{\prime },m^{\prime }}^{F}\left(f\right)}}, \end{array}$$

(5) $${\text{F-wPLI}}_{m,m^{\prime }}(f=\ell \nu_{0})\;≜\frac{\sum_{k=1}^{n_{j}}ℑ\{g_{X_{m}}(\ell ,k)g_{X_{m^{\prime }}}^{*}(\ell ,k)\}}{\sum_{k=1}^{n_{j}}\left|ℑ\{g_{X_{m}}(\ell ,k)g_{X_{m^{\prime }}}^{*}\left(\ell ,k\right)\right|\}}.$$

To quantify functional connectivity on MEG signals, the corresponding indices are practically computed as sums of the absolute values of these functions over the range of frequencies defining the targeted band-limited oscillations. Large values (above predefined thresholds) are used as markers of functional connectivity at the individual level, which are usually followed by statistical testing for assessing group-level significance.

### 2.2. Wavelet Domain Functional Connectivity Assessment

#### 2.2.1. Complex Wavelet Transform

The classical discrete wavelet transform relies on the use of a real-valued mother-wavelet (cf. e.g., Mallat, 1998). To assess phases and delays amongst signals, it is proposed here to use a complex wavelet transform, defined as follows. Let ψ^(*r*)^ denote a real-valued oscillating and sufficiently smooth reference pattern, referred to as the *mother wavelet*, and let it be constructed such that the collection of dilated and translated templates ${\left\{\psi_{j,k}\left(t\right)=2^{-j/2}\psi \left(2^{-j}t-k\right)\right\}}_{\left(j,k\right)\in {\mathbb{Z}}^{2}}$ of ψ form an orthonormal basis of *L*^2^(ℝ) (cf. e.g., Mallat, 1998). From ψ^(*r*)^, an analytic complex mother-wavelet can be defined as ψ = ψ^(*r*)^ + *1ψ*^(*1*)^, where ψ^(*1*)^ consists of the Hilbert transform of ψ^(*r*)^. The design of a complex, invertible, and analytic mother wavelet is not straightforward. In the present work, we build on the excellent approximate solution proposed in Kingsbury (2001) and Selesnick et al. (2005), which is referred to as the dual-tree complex wavelet transform.

For a signal *X*, the coefficients of the dual-tree complex wavelet transform are defined as $d_{X}\left(j,k\right)≜d_{X}^{\left(r\right)}\left(j,k\right)+1d_{X}^{\left(1\right)}\left(j,k\right)$, with $d_{X}^{\left(r\right)}\left(j,k\right)≜\int \psi_{j,k}^{\left(r\right)}\left(t\right)X\left(t\right)dt$ and $d_{X}^{\left(1\right)}\left(j,k\right)≜\int \psi_{j,k}^{\left(1\right)}X\left(t\right)dt$. Computing a dual-tree complex wavelet transform thus amounts to computing two standard Discrete Wavelet Transforms, with the two real mother-wavelets ψ^(*r*)^ and ψ^(*1*)^, respectively, independently.

#### 2.2.2. Wavelet Cross-Spectrum and Functional Connectivity

It has been well-documented that the study of univariate scale-free temporal dynamics should be performed using a wavelet-based spectral estimation rather than a Fourier-based one (cf. e.g., Flandrin, 1992; Abry and Veitch, 1998; Veitch and Abry, 1999, 2001). This has recently been extended to multivariate scale-free temporal dynamics analysis and wavelet cross-spectrum estimation (cf. e.g., Wendt et al., 2017; Abry and Didier, 2018b; La Rocca et al., 2018a; Abry et al., 2019b). Given a pair of signals *X*_*m*_, $X_{m^{\prime }}$, the multivariate wavelet (cross-)spectrum can be defined as

(6) $$\begin{array}{l} S_{m,m^{\prime }}^{W}\left(j\right)≜\frac{1}{n_{j}}\overset{n_{j}}{\underset{k=1}{\sum }}d_{X_{m}}\left(j,k\right)d_{X_{m^{\prime }}}^{*}\left(j,k\right) \end{array}$$

where $n_{j}\approx \frac{N}{2^{j}}$ are the number of coefficients available at scale *j*, and ^*^ stands for complex conjugate.

It has been shown (Abry et al., 2019b) that

(7) $$\begin{array}{l} 𝔼S_{m,m^{\prime }}^{\left(W\right)*}\left(j=\nu_{\psi }/2^{j}\right)=\int S_{m,m^{\prime }}\left(f\right)|\tilde{\psi }\left(f/2^{j}\right){|}^{2}df, \end{array}$$

with $\tilde{\psi }$ denoting the Fourier transform of ψ. This indicates that $S_{m,m^{\prime }}^{\left(W\right)}\left(j\right)$ estimates $S_{m,m^{\prime }}\left(f=\nu_{\psi }/a_{0}^{j}\right)$ around frequency $f=\nu_{\psi }/a_{0}^{j}$ and achieves a *fixed relative-frequency* resolution multivariate spectral analysis (Abry and Veitch, 1998; Abry et al., 2019b).

**Figure 1:**
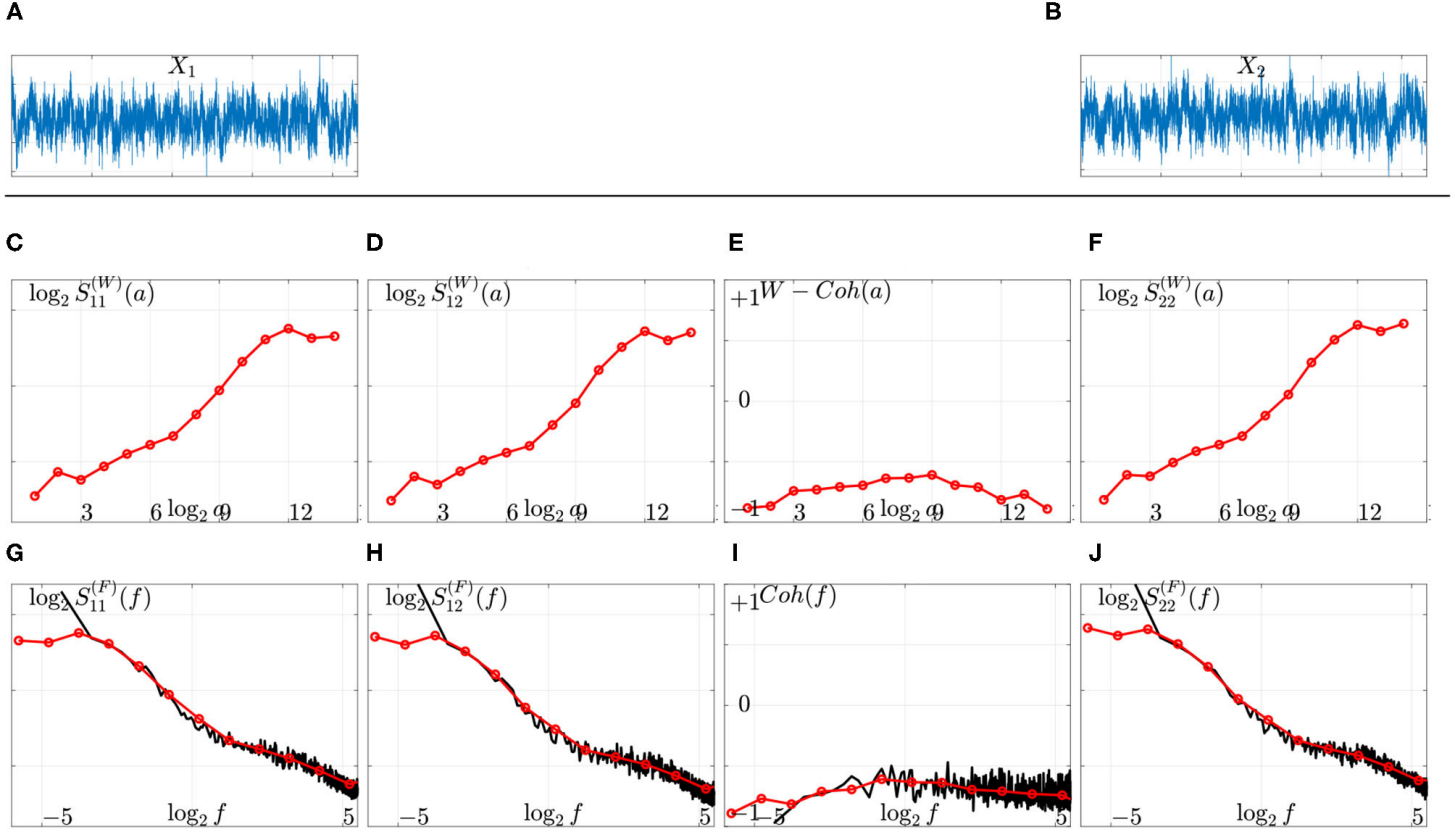
Fourier vs. wavelet spectral estimation on actual source-reconstructed MEG time series. Top: Two source-reconstructed MEG time series *X*_1_ **(A)** and *X*_2_ **(B)**. Middle: Wavelet spectra **(C,F)**, cross spectrum **(D)**, and coherence function **(E)** as functions of the (log of the) scales (top row, red lines). Bottom: Comparison to Fourier spectra **(G,J)**, cross-spectrum **(H)**, and coherence function **(I)** (solid black lines) after remapping scales into frequencies (bottom row). The scale-free (or arrhythmic) regime is marked by linear behaviors of the power spectra across coarse scales, 8 ≤ *j* ≤ 12 corresponding to low frequencies, 0.1 ≤ *f* ≤ 1.5 Hz, in these log log plots.

Equations (2) and (7) combined together show that Fourier-based $S_{m,m^{\prime }}^{\left(F\right)}$ and (Complex) Wavelet-based $S_{m,m^{\prime }}^{\left(F\right)}$ constitute two biased estimates of the power spectral density $S_{m,m^{\prime }}$, that can be compared theoretically and practically, as illustrated in Figure 1. Interested readers are referred to Abry and Veitch (1998) and Abry et al. (2019b) for further discussions. As an illustration, the wavelet spectra and cross-spectrum of the two MEG signals displayed in Figures 1A,B are shown in Figures 1C–F and compared to Fourier spectra and cross spectrum (cf. Figures 1G–J), using Equations (2) and (7) and converting scales *a* = 2^*j*^ into frequencies as $f=f_{0}\times f_{s}/2^{j}$, where *f*_*s*_ is the data sampling frequency and *f*_0_ a constant that depends on the specific choice of the mother wavelet. Readers interested by further theoretical and practical discussions on comparing Fourier and wavelet-based spectral estimations, are referred to e.g., Abry and Veitch (1998), Veitch and Abry (1999), Veitch and Abry (2001), Abry et al. (2000), Ciuciu et al. (2012), and Abry et al. (2019b).

#### 2.2.3. Wavelet-Based Functional Connectivity Indices

From the wavelet-based estimate of the power spectrum, wavelet-based indices can be constructed to assess functional connectivity, as was the case with Fourier spectrum and mutatis mutandis:

(8) $$\begin{array}{l} {\text{W-COH}}_{m,m^{\prime }}\left(j\right)≜\frac{S_{m,m^{\prime }}^{W}\left(j\right)*}{\sqrt{S_{m,m}^{W}\left(j\right)S_{m^{\prime },m^{\prime }}^{W}\left(j\right)*}}, \end{array}$$

(9) $$\begin{array}{l} {\text{W-ICOH}}_{m,m^{\prime }}\left(j\right)≜\frac{ℑ\{S_{m,m^{\prime }}^{W}\left(j\right)\}}{\sqrt{S_{m,m}^{W}\left(j\right)S_{m^{\prime },m^{\prime }}^{W}\left(j\right)\}}}, \end{array}$$

(10) $$\begin{array}{l} {\text{W-wPLI}}_{m,m^{\prime }}\left(j\right)≜\frac{\sum_{k=1}^{n_{j}}ℑ\{d_{X_{m}}\left(j,k\right)d_{X_{m^{\prime }}}^{*}\left(j,k\right)\}}{\sum_{k=1}^{n_{j}}|ℑ\{d_{X_{m}}\left(j,k\right)d_{X_{m^{\prime }}}^{*}\left(j,k\right)\}|}. \end{array}$$

Unlike the standard discrete wavelet transform coherence function used in, e.g., Whitcher et al. (2000) and Wendt et al. (2017), ${\text{W-COH}}_{m,m^{\prime }}\left(j\right)$ is *complex-valued*.

#### 2.2.4. Functional Connectivity Assessed From Fractal Dynamics

Functional connectivity for scale-free infraslow temporal dynamics consists of averaging the absolute values of these functions over the corresponding range of octaves *j*_1_ ≤ *j* ≤ *j*_2_ (equivalently over the range of scales *a* = 2^*j*^ or frequencies $f=f_{0}/2^{j}$) where scale-free dynamics are observed:

$$\frac{1}{j_{2}-j_{1}+1}\overset{j_{2}}{\underset{j=j_{1}}{\sum }}{\text{W-wPLI}}_{m,m^{\prime }}\left(j\right)\text{ or }\frac{1}{j_{2}-j_{1}+1}\overset{j_{2}}{\underset{j=j_{1}}{\sum }}{\text{W-ICOH}}_{m,m^{\prime }}\left(j\right)$$

**Figure 2:**
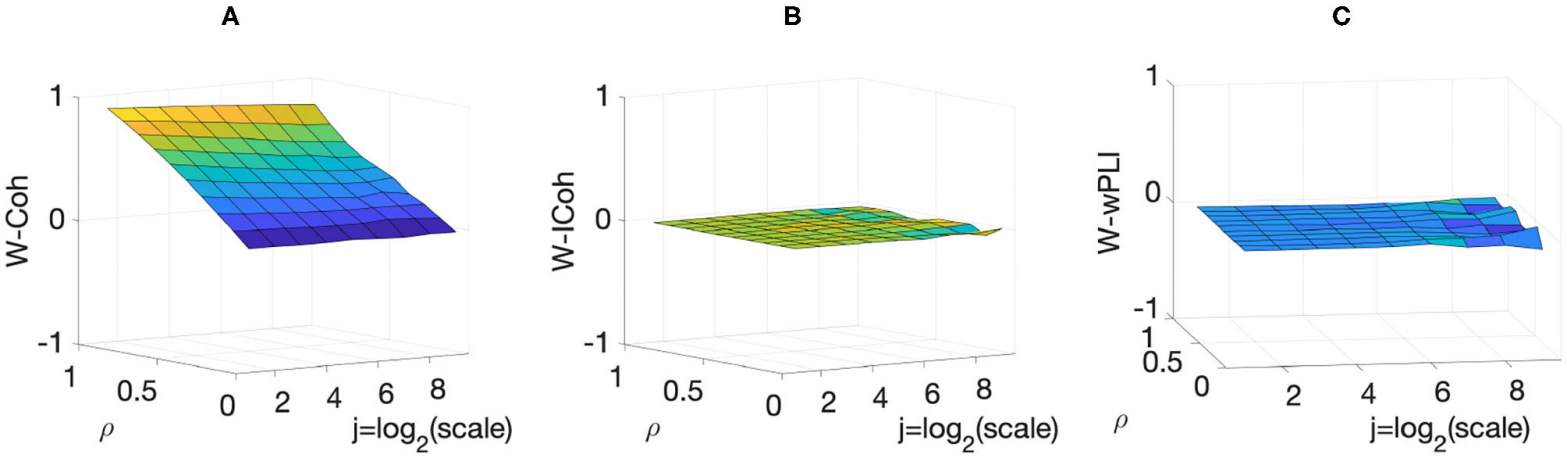
Complex Wavelet-based connectivity on synthetic bivariate fractional Gaussian noise with correlation but no delay. W-COH **(A)**, W-ICOH **(B)**, and W-wPLI **(C)** as function of octaves *j* and correlation coefficient ρ. As it should, W-COH correctly assesses correlations with no delays and thus departs from 0 at all scales. W-COH would hence lead to incorrectly assessing functional connectivity. In contrast, W-ICOH and W-wPLI show averages values of 0 at all scales and across all correlation levels, thus leading to assess no connectivity, which is as expected for non-delayed components.

**Figure 3:**
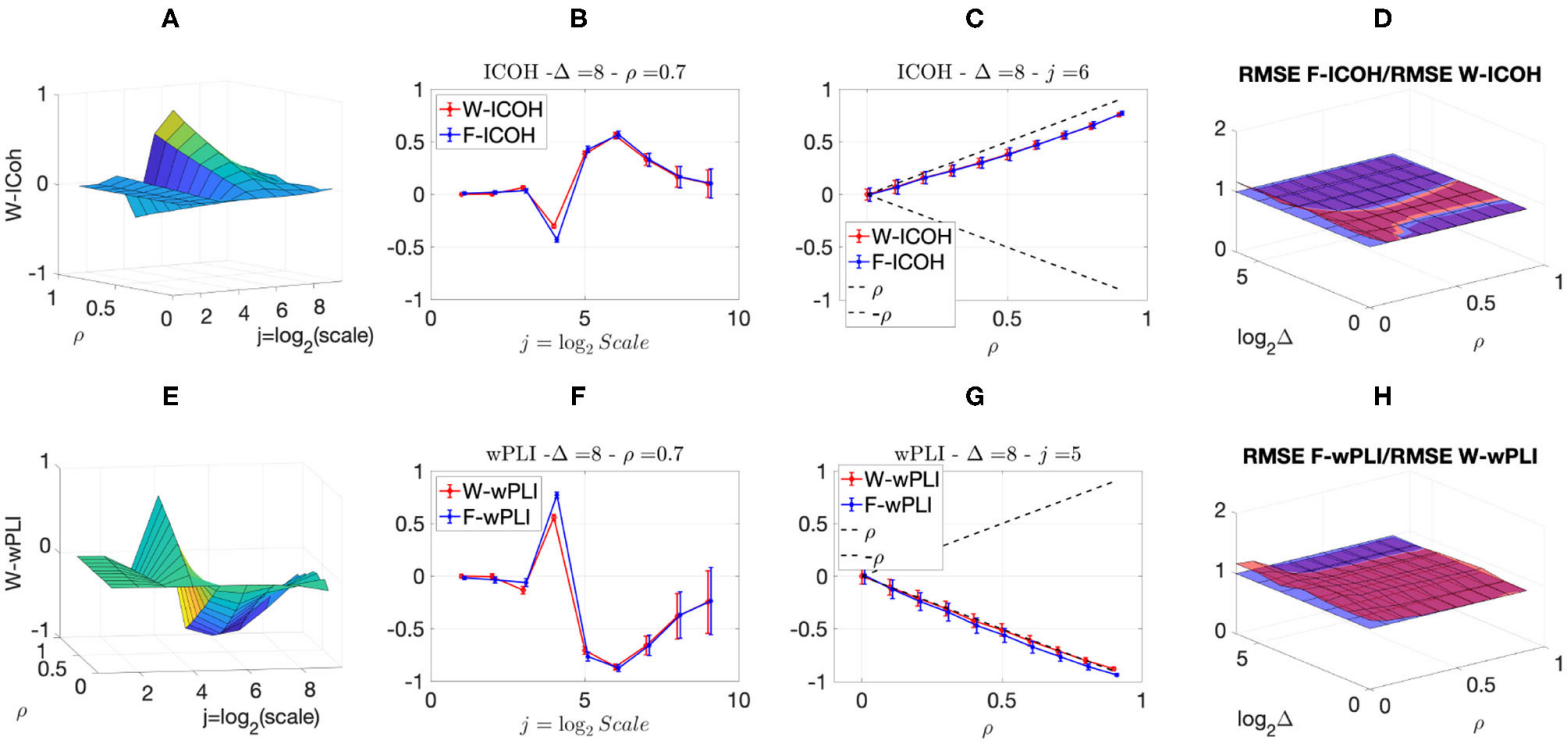
Complex Wavelet-based connectivity on synthetic bivariate fractional Gaussian noise with correlation and delay Δ = 8. Top row: W-ICOH results. Bottom row: W-wPLI results. From left to right: W-ICOH **(A)** and W-wPLI **(E)** as functions of octaves *j* and correlation coefficient ρ; W-ICOH **(B)** and W-wPLI **(F)** as functions of octaves *j*, for a given ρ; W-ICOH **(C)** and W-wPLI **(G)** as functions of ρ for given octaves *j*; Ratio of the RMSE of F-ICOH to W-ICOH **(D)** and ratio of RMSE of F-wPLI to W-wPLI **(H)**, averaged across scales 3 ≤ *j* ≤ 7, and color-coded in red as functions of delay Δ and correlation coefficient ρ. A ratio larger than the value of 1 (made explicit to ease comparisons by horizontal blue plans) indicates poorer performance for Fourier-based estimates compared to wavelet-based ones. Synthetic data consists of bivariate fGn with *H*_1_ = 0.7 and *H*_2_ = 0.8.

**Figure 4:**
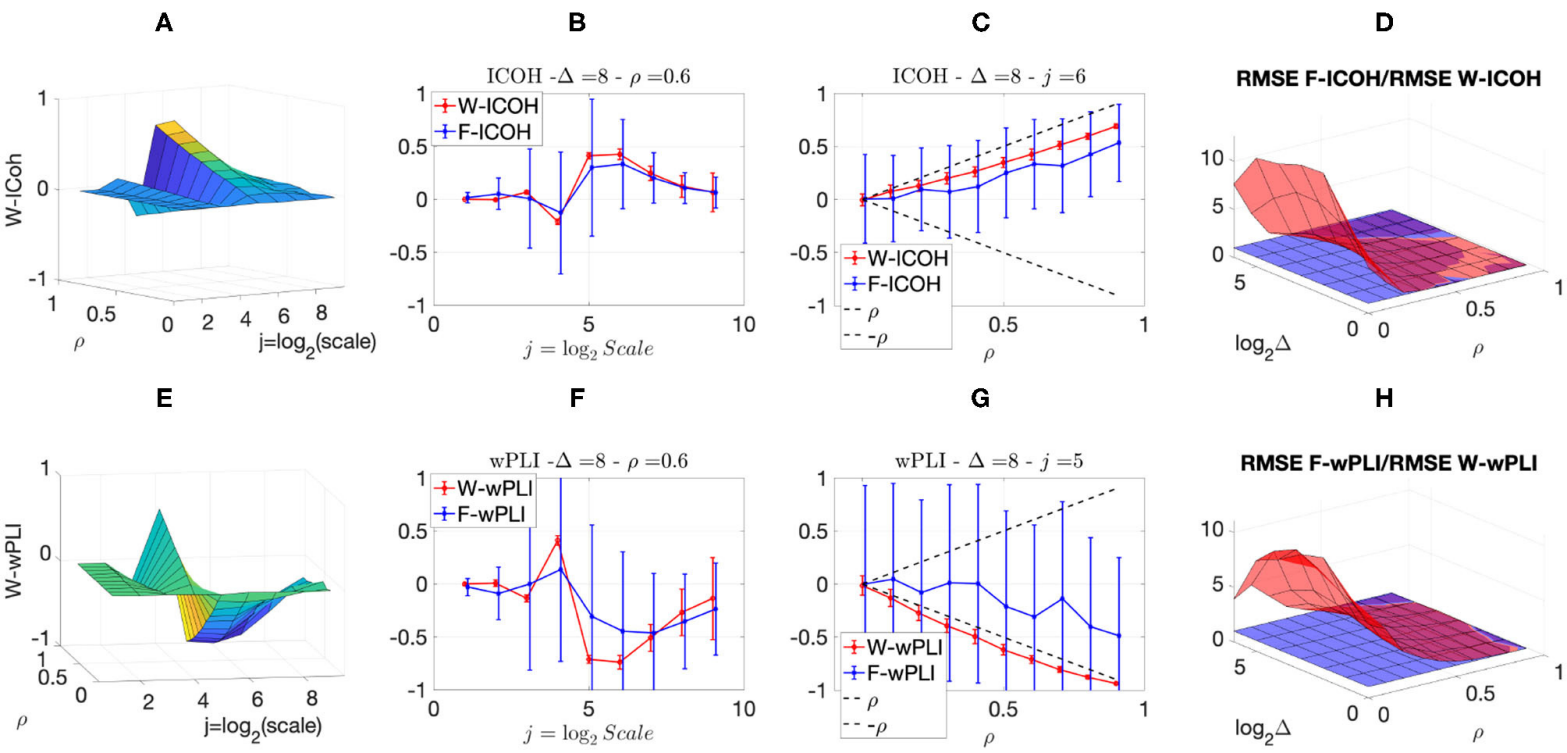
Complex Wavelet-based connectivity on synthetic bivariate fractional Brownian motion with correlation and delay Δ = 8. Top row: W-ICOH results. Bottom row: W-wPLI results. From left to right: W-ICOH **(A)** and W-wPLI **(E)** as functions of octaves *j* and correlation coefficient ρ; W-ICOH **(B)** and W-wPLI **(F)** as functions of octaves *j*, for a given ρ; W-ICOH **(C)** and W-wPLI **(G)** as functions of ρ for given octaves *j*; Ratio of the RMSE of F-ICOH to the RMSE of W-ICOH **(D)** and ratio of the RMSE of F-wPLI to the RMSE of W-wPLI **(H)**, averaged across scales 3 ≤ *j* ≤ 7, and color-coded in red as functions of delay Δ and correlation coefficient ρ. A ratio greater than the value of 1 (made explicit to ease comparisons by horizontal blue plans) indicates poorer performance for Fourier-based estimates compared to wavelet-based ones. Synthetic data consists of bivariate fBm with *H*_1_ = 0.7 and *H*_2_ = 0.8.

Remapping scales into frequencies, calculations inspired from those leading to Equations (2) and (7) permit to compare theoretically and practically W-COH, W-ICOH and W-wPLI to F-COH, F-ICOH, and F-wPLI, as illustrated in Figures 2–**4** on synthetic data.

This is here critical to emphasize that *functional connectivity assessed from fractal dynamics* as defined and used in the present work is associated with (the statistics of) cross-temporal dynamics. It should not be confused with the so-called *fractal networks*, as studied in, e.g., in Bassett et al. (2006) and Varley et al. (2020), which are related to topological (thus static) properties of a spatial graph.

### 2.3. Functional Connectivity From Fractal Dynamics Performance Assessment

#### 2.3.1. Monte Carlo Numerical Simulations

To assess the performance of the proposed indices aiming to quantify functional connectivity from fractal dynamics, Monte Carlo numerical simulations were conducted. They make use of synthetic bivariate fractional Brownian motion, a specific instance of the multivariate selfsimilarmodel recently introduced in Didier and Pipiras (2011) and studied in Abry and Didier (2018a,b). Bivariate fractional Brownian motion consists of a pair of fractional Brownian motions *B*_*H*_1__ and *B*_*H*_2__, with possibly different selfsimilarity parameters *H*_1_ and *H*_2_, with pointwise correlation ρ. In addition, one component is delayed by Δ. Correlation coefficient ρ is set to range within ρ ∈ {0, 0.1, 0.2, 0.3, 0.4, 0.5, 0.6, 0.7, 0.8, *and* 0.9} and delays range in Δ = {0, 1, 2, 4, 8, 16, 32, *and* 64} samples. Sample size is *n* = 2^14^, chosen to match the size of the infraslow regime of the MEG data (cf. sections 3 and 4).

To model MEG data as those analyzed in section 4 and as commonly indicated in the literature (He et al., 2010), one needs to use both fractional Gaussian noise (fGn), the increments of fractional Brownian motion (fBm), with parameter *H* ranging from say 0.6 to 1 and fractional Brownian motion itself with parameters ranging from 0 to 1. Therefore, the numerical simulations conducted here were based on bivariate processes, each component being either fGn or fBm, with 0 < *H* < 1. For the Fourier-based spectral estimation, the classical averaged windowed periodogram estimate of the power spectral density was computed, with Hanning windows of a width corresponding to the frequency bands of the complex wavelet filters, to enable relevant comparisons of the tools. For the Complex-Wavelet based estimation, q-shift complex wavelets were used, as described in Selesnick et al. (2005) and references therein (see, e.g., Lina and Mayrand, 1995 for an alternative choice).

Indices assessing functional connectivity from fractal dynamics (both Fourier and wavelet-based) were computed as average over a range of frequencies and scales that match those of the infraslow scale-free range observed on the MEG data described and analyzed hereafter. Performances are reported as means (and confidence intervals) computed from *N* = 1, 000 independent realizations of bivariate fractional Gaussian noise.

#### 2.3.2. Spurious Connectivity

To start with, we analyzed scenarios where the two components of bivariate fractional Gaussian noise were correlated but not delayed: Δ ≡ 0. Figure 2 reports the averaged (over realizations) values of W-COH, W-ICOH, and W-wPLI as functions of octaves *j* and correlation coefficients ρ. Figure 2A shows that W-COH correctly assesses correlations between components as predicted by theory when they are not delayed. W-COH thus leads to an incorrect assessment of functional connectivity since it is sensitive to 0-delay correlation and thus to the volume conduction effect. This spurious connectivity consists of a well-documented fact for the classical (Fourier-based) coherence function index F-COH, which is, as theoretically expected, not corrected by the use of W-COH. Figures 2B,C also shows that W-ICOH and W-wPLI average to 0 at all scales, and across all correlation levels, thus correctly leading to the assessment of no functional connectivity, as expected for non-delayed components. Again, this is consistent with observations made when using the Fourier-based F-ICOH and F-wPLI. This rules out the use of W-COH (and F-COH) to assess functional connectivity.

#### 2.3.3. Functional Connectivity Assessed From Fractal Dynamics

We then analyzed signals with delays amongst components. Figures 3, 4 report, for different sets of synthetic data and for given delays Δ, the averaged values (over realizations) of W-ICOH and W-wPLI as functions of octaves *j* and correlation coefficients ρ [left column, see panels (A) and (E)], complemented with slices for fixed ρ as functions of *j* [second column, see panels (B) and (F)], slices for fixed *j* as functions of ρ [third column, see panels (C) and (G)], and functional connectivity indices averaged across scales 3 ≤ *j* ≤ 7 [right column, see panels (D) and (H)]. Figures 3, 4 show that:

(i) Both W-ICOH and W-wPLI do depart from 0 across *j* and ρ when Δ ≠ 0 (left column).

(ii) As functions of *j*, W-ICOH and W-wPLI display different patterns that depend on Δ. However, these patterns both show independently maximum absolute deviations from 0 at scales that vary with Δ (second column). This was quantified for W-ICOH and used as a delay estimation procedure (Didier et al., 2019).

(iii) When a scale 2^*j*^ in relation to Δ is chosen, both (the absolute values of) W-ICOH and W-wPLI are proportional to (the absolute value of) ρ (third column). This shows not only that W-ICOH and W-wPLI depart from 0 when delays amongst components exist but also that the amplitude of the departure is proportional to the correlation ρ between components, a crucial property to assess quantitatively functional connectivity, clearly and originally quantified in these numerical simulations.

(iv) The conclusions stemming from comparing the performance of Fourier-based F-ICOH and F-wPLI to Complex Wavelet-based W-ICOH and W-wPLI depend on the parameters used for simulating bivariate synthetic time series. When the latter consist of bivariate fGn with *H*_1_ = 0.7 and *H*_2_ = 0.8 (Figure 3), F-ICOH vs. W-ICOH and F-wPLI vs. W-wPLI, show comparable performance either in bias (second and third columns) or in terms of root mean square error (RMSE) (right column). When synthetic data consists of bivariate fBm with *H*_1_ = 0.7 and *H*_2_ = 0.8 (Figure 4), F-ICOH and F-wPLI show significantly degraded performance compared to W-ICOH and W-wPLI, both in bias and variance (second and third columns) and in terms of RMSE (right column). Notably, RMSE of F-ICOH and F-wPLI can be 10 times greater than RMSE of W-ICOH and W-wPLI for small values of ρ. Complex Wavelet-based indices thus outperform Fourier-based ones for data with large scaling exponents, i.e., large powers at very low frequencies or, in other words, very slow dynamics. Similar conclusions can be drawn from other values of delays Δ ≠ 0 tested here but not shown (available upon request).

#### 2.3.4. Functional Connectivity Assessed From Fractal Dynamics in the Presence of Additive Trends

**Figure 5:**
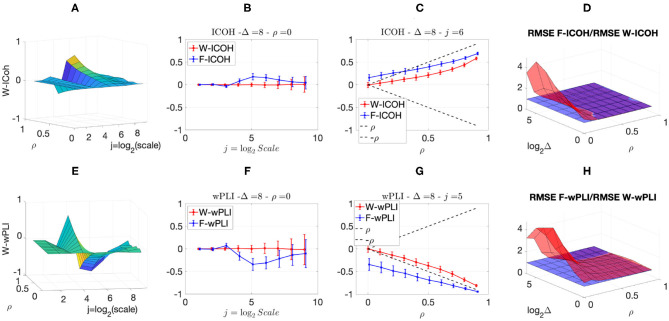
Complex Wavelet-based connectivity on synthetic bivariate fractional Gaussian noise with correlation and delay, and additive trends. Top row: W-ICOH results. Bottom row: W-wPLI results. From left to right: W-ICOH **(A)** and W-wPLI **(E)** as functions of octaves *j* and correlation coefficient ρ, W-ICOH **(B)** and W-wPLI **(F)** as functions of octaves *j*, for a given ρ, and W-ICOH **(C)** and W-wPLI **(G)** as functions of ρ for given octaves *j*. The ratio of the RMSE of F-ICOH to the RMSE of W-ICOH **(D)** and the ratio of the RMSE of F-wPLI to the RMSE of W-wPLI **(H)**, averaged across scales 3 ≤ *j* ≤ 7 and color-coded in red as functions of delay Δ and correlation coefficient ρ. A ratio greater than the value of 1 (made explicit to ease comparisons by horizontal blue plans) indicates poorer performance for Fourier-based estimates compared to wavelet-based ones. Synthetic data consists of bivariate fGn with *H* = 0.8 and fBm with *H* = 0.2.

We finally analyzed more complicated scenarios with correlation and delays amongst components as well as additive smooth slow trends superimposed as noise to the actual scale-free components. Figure 5 reports, for a given delay Δ = 8, the averaged (over realizations) values of W-ICOH and W-wPLI as functions of octaves *j* and correlation coefficient ρ [left column, panels (A) and (E)], complemented with slices for fixed ρ as functions of *j* [second column, panels (B) and (F)] and slices for fixed *j* as functions of ρ [third column, panels (C) and (G)]. Focusing the analysis of Figure 5 on ρ = 0 or on the small values of ρ shows the following:

(i) F-ICOH and F-wPLI depart from 0 across scales when there is no correlation while the Complex Wavelet-based W-COH and W-wPLI do not (second column);

(ii) F-ICOH and F-wPLI significantly overestimate correlations at small ρ while W-COH and W-wPLI do not (third column);

(iii) The RMSE of F-ICOH and F-wPLI becomes up to ten times larger than RMSE of W-ICOH and W-wPLI for small values of ρ (fourth column).

#### 2.3.5. Functional Connectivity From Fractal Dynamics Assessment Performance

**Figure 6:**
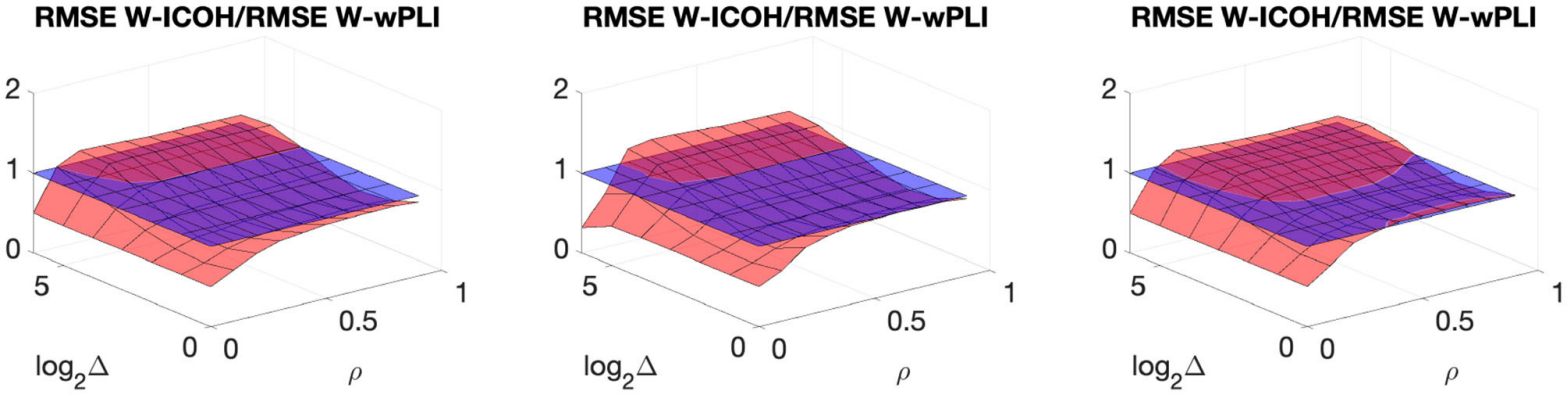
Ratio of the RMSE of W-ICOH to the RMSE of W-wPLI, averaged across scales 3 ≤ *j* ≤ 7, as functions of delay Δ and correlation coefficient ρ, for the synthetic data in Figures 3–5. Horizontal blue plans indicate the constant level of 1 to ease reading.

In addition, Figure 6 compares the ratio of the RMSE of W-ICOH to the RMSE of W-wPLI over several synthetic data sets and shows that both indices perform comparably. However, W-ICOH shows a slightly smaller RMSE for small values of ρ and conversely, a slightly larger RMSE for large values of ρ and for the largest delays Δ tested here. This (slight) superiority of W-wPLI is much more visible when additive smooth trends are present (right plot). In sum, these numerical simulations yield the following conclusions for the assessment of functional connectivity from fractal dynamics.

(i) They indicate that W-COH cannot be used to assess functional connectivity as it is fooled by zero-delay (volume conduction effect) correlations, thus confirming an already documented observation for F-COH in the literature (Nolte et al., 2004; Stam et al., 2007). To the converse, W-ICOH and W-wPLI (and F-ICOH and F-wPLI) are much less affected by these spurious correlations.

(ii) The Complex Wavelet W-ICOH and W-wPLI can be used to assess functional connectivity for scale-free temporal dynamics.

(iii) The Complex Wavelet W-ICOH and W-wPLI perform significantly better than the Fourier-based F-COH and F-wPLI first when the signals show very large scaling exponents β in their *f*^−β^ power spectral density behavior, as is the case with fBm-like time series and second when additive noise in the form of smooth and slow trends are superimposed to data with scale-free dynamics, which is a situation commonly observed in recordings collected from neuroimaging techniques.

(iv) W-ICOH and W-wPLI perform comparably with (slightly) better performance of W-wPLI when ρ or Δ increases, or when smooth trends are superimposed to scale-free dynamics, as often the case on MEG data. This will be further discussed in section 4.

## 3. Experimental MEG Data

The proposed complex wavelet-based assessment of functional connectivity in infraslow arrhythmic brain activity was tested on MEG measurements, consisting of non-invasive recordings of simultaneous time-series reflecting the whole brain activity, both at rest and during the completion of a task. All details about the experimental paradigm and the task can be found in Zilber et al. (2014).

In short, the task was designed from a short-term learning paradigm and consisted of visual coherence discrimination. Two sets of colored (green and red) dots were mixed and shown on a screen, each dot with random and independent movement. After a variable duration interval (0.3–0.6 s) of incoherent motion, a fraction of randomly chosen dots belonging to either of the two sets (also randomly chosen at each trial) followed a coherent motion during 1 s. Participants were asked to tell which of the red or green clouds had a coherent motion by pressing a button of the same color. Task difficulty was increased by decreasing the rate of dots in coherent motion.

The experiment was organized as interleaved MEG blocks alternating rest and task measurements: It started with a 5-min rest recording (REST_*i*_), followed by a 12-min pre-training block (TASK_*i*_); this was followed by four successive 5-min long individualized training blocks. Another 5-min resting-state block (REST_*f*_) was recorded prior to a final 12-min post-training block (REST_*f*_), consisting of the same visual coherence discrimination task as in TASK_*i*_. During TASK_*i*_ and TASK_*f*_, the motion coherence discrimination accuracy of each participant was assessed. Pre-training and post-training behavioral thresholds were computed for each participant as the visual coherence level associated with 75% of correct responses (hit rate). During REST blocks, participants were instructed to keep their eyes open, and they were not following any other explicit instruction, thus permitting the analysis of spontaneous fluctuations of brain activity from MEG recordings.

For the experiment, 36 healthy participants (mean age: 22.1 ± 2.2) were recruited. All participants were right-handed, had normal hearing, and had normal or corrected-to-normal vision. Before the experiment, all participants provided written informed consent in accordance with the Declaration of Helsinki (2008) and the local Ethics Committee on Human Research at NeuroSpin (Gif-sur-Yvette, France).

Brain activity was recorded via MEG modality in a magnetically shielded room using a 306 MEG system (Neuromag Elekta LTD, Helsinki). MEG signals originally sampled at 2 kHz were downsampled at 448 Hz and preprocessed to remove external and internal interferences in accordance with accepted guidelines for MEG research (Gross et al., 2013). Signal Space Separation (SSS) was applied with MaxFilter to remove exogenous artifacts and noisy sensors (Taulu and Simola, 2006). Ocular and cardiac artifacts (eye blinks and heartbeats) were removed using Independent Component Analysis (ICA) on raw signals. ICA were fitted to raw MEG signals, and sources matching the ECG and EOG were automatically found and removed before signals reconstruction, following the procedure described in Jas et al. (2017). Source localization from MEG signals was used to reconstruct source cortical activity using the mne_analyze tools within MNE (Gramfort et al., 2013). Details regarding the source localization technique are reported in Zilber et al. (2014). Finally, following analyses reported in Zilber et al. (2014) and La Rocca et al. (2020), 28 cortical regions-of-interest (ROIs), recruited in task performance (including frontal, somato-sensory, temporal, parietal, and occipital areas) were retained for the analysis of functional connectivity in infraslow temporal dynamics.

## 4. Functional Connectivity Assessed From Fractal Dynamics in Infraslow Arrhythmic MEG-Recorded Brain Activity

### 4.1. Infraslow Scale/Frequency Range and Functional Connectivity From Fractal Dynamics Assessment Methodology

#### 4.1.1. Infraslow Scale/Frequency Range

Following the systematic inspections of the wavelet spectra and cross-spectra reported in La Rocca et al. (2018b) for the same MEG data, the scale-free range of scales is set uniformly for the 28 times series and across the 36 participants for the analysis of arrhythmic functional connectivity to 8 ≤ *j* ≤ 12, thus corresponding to frequencies in 0.1 ≤ *f* ≤ 1.5 Hz or equivalently to time scales ranging roughly from 1 to 10 s. This scale-free regime is illustrated in Figure 1 for arbitrarily chosen MEG signals shown in Figures 1A,B.

#### 4.1.2. Experimental Conditions

Infraslow functional connectivity was assessed for several experimental conditions: resting-state (REST_*i*_), pre-training (TASK_*i*_), and post-training (TASK_*f*_) tasks, thus enabling us to assess changes in functional interactions from rest to task and modulations related to learning.

#### 4.1.3. Functional Connectivity From Fractal Dynamics Indices

Three proposed complex wavelet based indices were then computed to assess infraslow functional connectivity by averaging across octaves corresponding to the scale-free regime, 8 ≤ *j* ≤ 12, and the functions W-COH(*j*), W-ICOH(*j*), and W-wPLI(*j*), resulting in three sets of 28 × 28 × 36 indices.

##### 4.1.3.1. Tests

These indices were filtered at the group-level (*N* = 36), using a recently introduced network density threshold method, the Efficiency Cost Optimization (De Vico Fallani et al., 2017), thus yielding group-level 28 × 28 fractal dynamics-based functional connectivity matrices across the brain for each experimental condition independently. See also La Rocca et al. (2020) for further details on the use of such technique.

To investigate significant differences in infraslow functional connectivity between two different experimental conditions (e.g., TASK_*i*_ − REST_*i*_) independently for each chosen index, a group-level paired *t*-test was performed, with a demanding preset significance level: *p* < 0.01. The false discovery rate (FDR) procedure was used to correct *p*-values for multiple comparisons across the 28 × 27/2 possible connections.

##### 4.1.3.2. Comparisons Against Fourier-Based Indices

To compare Fourier-based F-ICOH and F-wPLI to Complex Wavelet-based W-ICOH to W-wPLI, Fourier-based spectral estimation was conducted using Welch Periodogram procedures (as described in section 2.1), using a windowed Fourier transform with a Hanning-type window of duration 80s.

### 4.2. Fractal Dynamics-Based Functional Connectivity Networks

**Figure 7:**
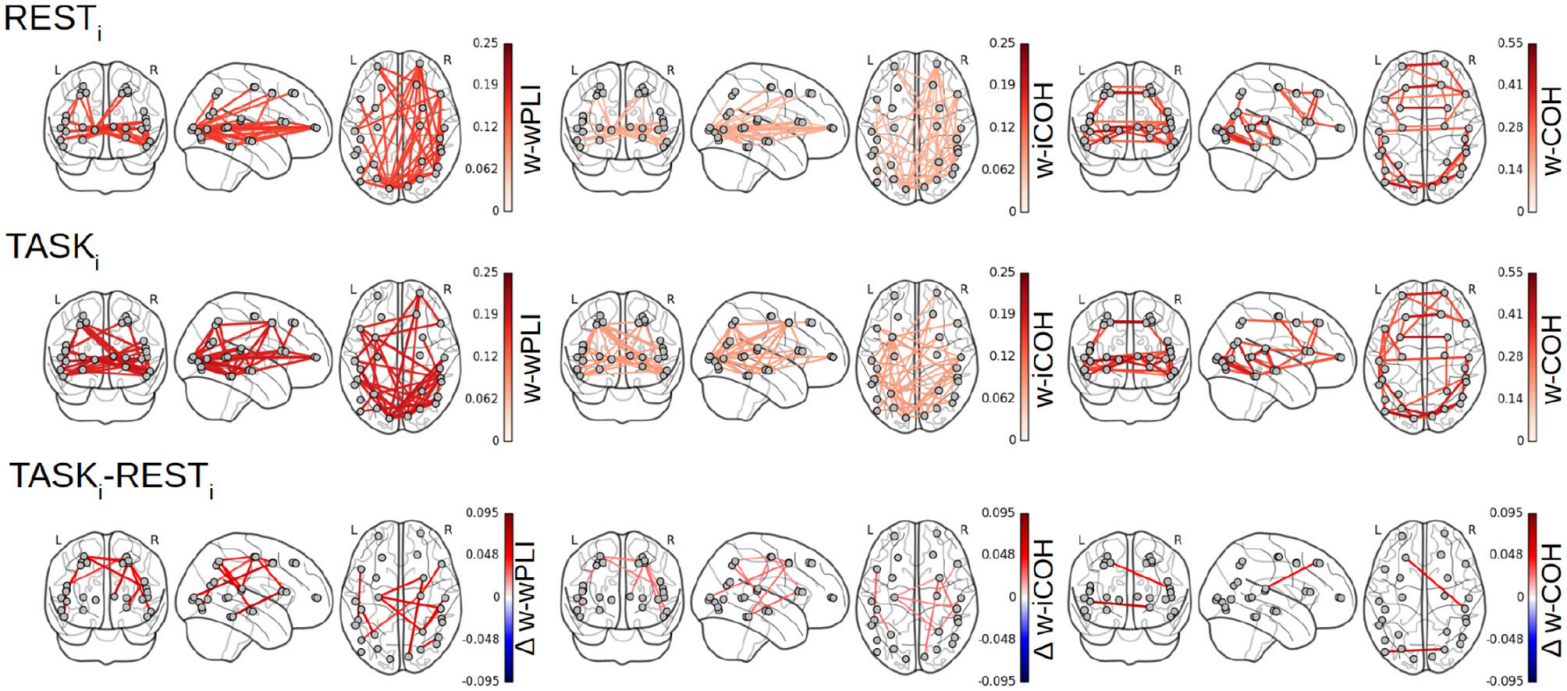
Functional connectivity assessment from fractal dynamics: Group-level functional connectivity in infraslow MEG-source reconstructed brain dynamics. Filtered 28 × 28 connectivity networks measured from Complex Wavelet based W-wPLI (left), W-ICOH (middle), and W-COH (right), for REST_*i*_ (top row) and pre-training TASK_*i*_ (center row). The red color intensity codes for the values of the connectivity indices (ranging from 0 to 1 by construction). Functional connectivity differences between conditions TASK_*i*_ and REST_*i*_ when assessed as significant by a group level FDR corrected *t*-test are displayed in the bottom row. Color codes for the TASK_*i*_ − REST_*i*_ differences in the values of indices from blue (negative) to red (positive), thus indicating that only increases in functional connectivity are observed from REST_*i*_ to TASK_*i*_.

Figure 7 reports the 28 × 28 thresholded connectivity networks yielded by the Complex Wavelet based indices defined in section 2, W-wPLI (left), W-ICOH (middle), and W-COH (right), for two different experimental conditions REST_*i*_ (top row) and pre-training TASK_*i*_ (center row). Further, Figure 7 (bottom row) reports the FDR-corrected statistically significant differences between indices measured during TASK_*i*_ and REST_*i*_. Figure 7 leads to the following observations:

(i) The connectivity networks yielded by W-COH predominantly display short-range and inter-hemispheric interactions throughout the cortex and most notably amongst frontal regions on one hand and temporo-occipital regions on other hand, both for REST_*i*_ and TASK_*i*_.

(ii) The connectivity networks yielded by W-ICOH and W-wPLI display similar structures, dominated by long-range spatial interactions, that differ significantly from those of the networks produced by W-COH, dominated by shorter-range spatial interactions. These differences in network structures can be quantified using the Average Degree, i.e., the average number of connections per node, as a network structure metrics. For REST_*i*_, the Average Degrees for the graphs obtained by W-COH, W-ICOH, and W-wPLI are of 0.95(±0.37), 0.21(±0.24), and 0.44(±0.52), respectively. Medians distributions of the number of links per node differ significantly between W-COH and W-ICOH (*p* < 10^−11^) or between W-COH and W-wPLI (*p* < 10^−6^). The same holds for TASK_*i*_, with average degrees of 1.0(±0.49), 0.25(±0.24), and 0.52(±0.50), respectively, and significances of *p* < 10^−8^ and *p* < 10^−3^, respectively.

(iii) While yielding comparable networks, W-wPLI and W-ICOH differ insofar as the former yields larger connectivity indices than the latter. In addition, connectivity networks using W-wPLI or W-ICOH differ in structure; however, they differ much less than when comparing W-wPLI vs. W-COH or W-ICOH vs. W-COH. Indeed, for REST_*i*_ the Average Degrees of W-wPLI and W-ICOH are 0.44(±0.52) and 0.21(±0.24), respectively, yielding a quantifiable difference (*p* = 0.04), and for TASK_*i*_ the Average Degrees of W-wPLI and W-ICOH are 0.52(±0.50) and 0.25(±0.24), respectively, yielding a clearer difference (*p* = 0.01).

(iv) When comparing TASK_*i*_ vs. REST_*i*_, W-wPLI and W-ICOH both indicate an increase in functional connectivity during task performance. This increase in functional connectivity assessed from fractal dynamics highlights interactions between regions recruited in the achievement of the task, notably fronto-temporal couplings [between the right ventro-lateral prefrontal cortex (vlPFC) and inferior temporal cortex (ITC)], interactions linking temporal regions [anterior superior temporal sulcus (aSTS) and auditory cortex] with the intra-parietal sulcus (IPS), motor-occipital couplings between the left frontal BA6 (including premotor and supplementary motor regions), and primary visual areas (V1/V2). Interaction between the key region hMT+, sensitive to visual motion, and the associative area, pSTS, is also significant in the left hemisphere.

**Figure 8:**
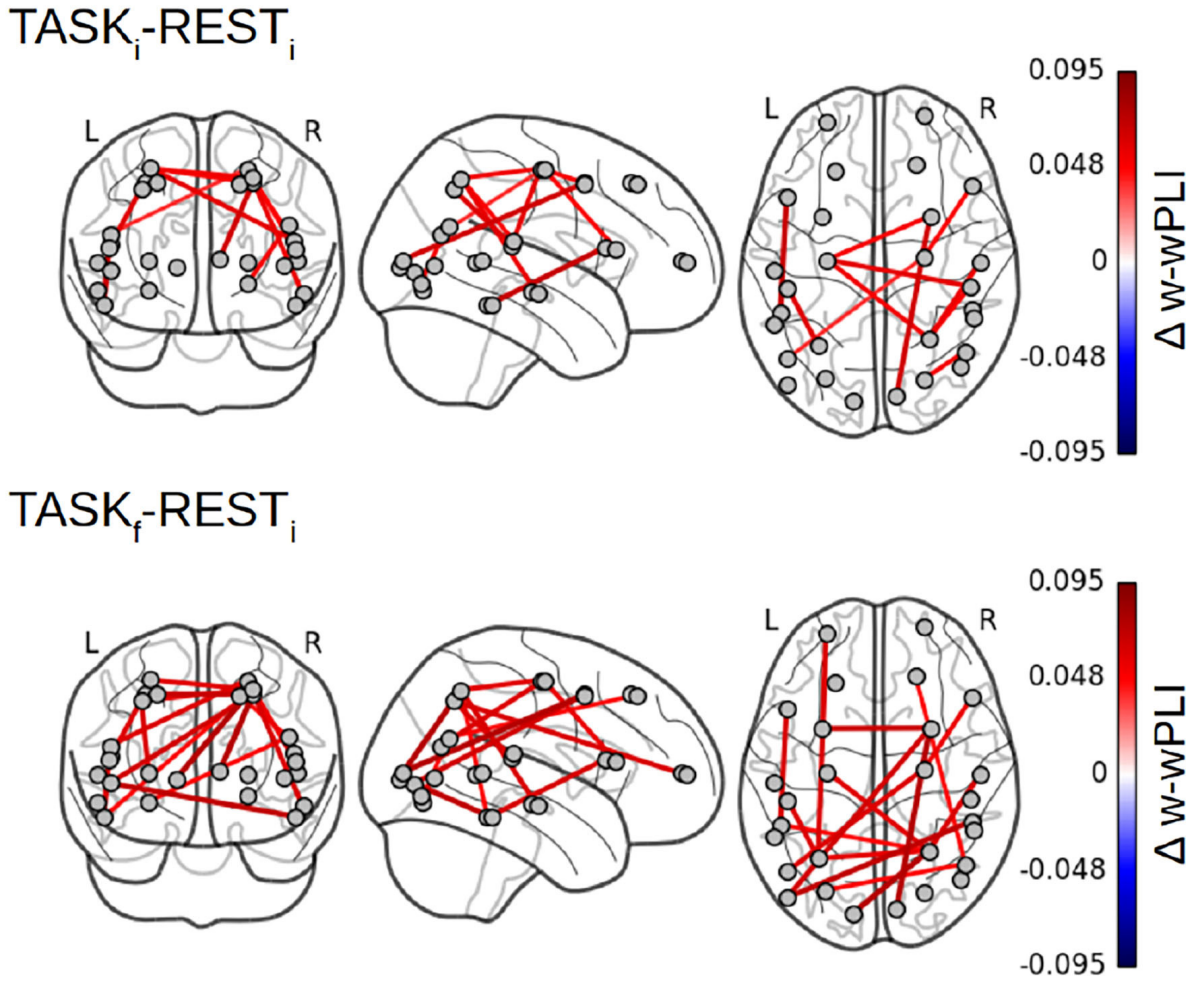
Fractal dynamics-based functional connectivity assessment (W-wPLI) differences between REST_*i*_ and TASK_*i*_ and between REST_*i*_ and TASK_*f*_. The increase in functional connectivity assessed from fractal dynamics from rest to task is strengthened with training, i.e., from TASK_*i*_ to TASK_*f*_, and emerged between several intra- or inter-hemispheric pairs of regions (Frontal polar/IPS, ITC/MT, FEF/pSTS) involved in task performance.

Focusing on the W-wPLI index only, Figure 8 shows the additional comparisons of the post-training task TASK_*f*_ to the initial rest REST_*i*_, which, compared to the contrast TASK_*i*_ − REST_*i*_ (cf. Figure 7 bottom left plot), indicates first that functional interactions in infraslow temporal dynamics are globally strengthened by the training and second that new intra- and inter-hemispheric couplings emerged with training involving much more the parieto-occipito-temporal network (IPS, primary visual cortex, and anterior STS). We also noticed new interactions between the left fronto-polar region and the left IPS, the right frontal eye fields (FEF) and the pSTS, and the BA6 complex and hMT+ region.

### 4.3. Functional Connectivity Assessed From Fractal Dynamics and Selfsimilarity

**Figure 9:**
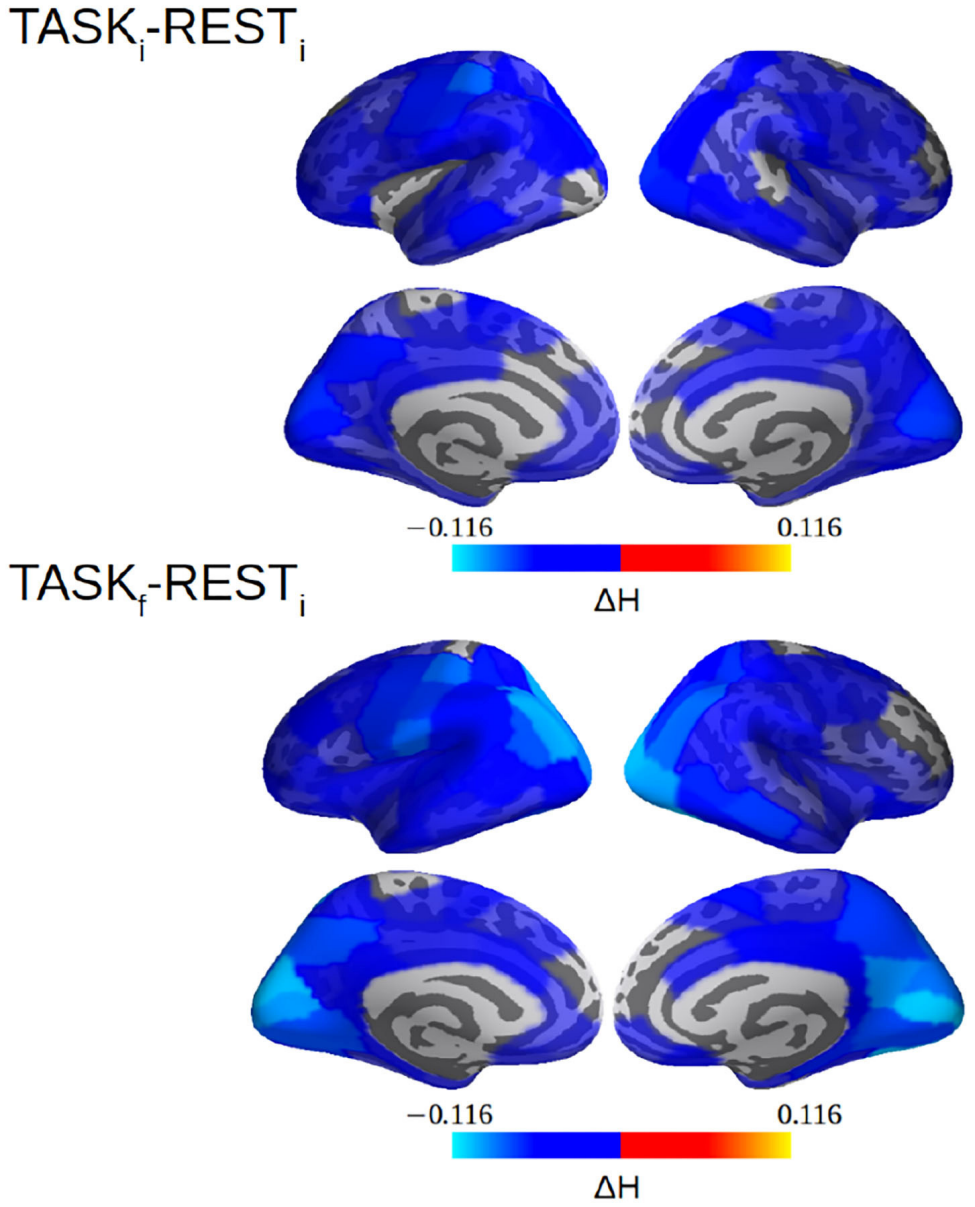
selfsimilarity (*H*) differences between REST_*i*_ and TASK_*i*_ and between REST_*i*_ and TASK_*f*_. The decrease in selfsimilarity from rest to task is strengthened with training, i.e., from TASK_*i*_ to TASK_*f*_, and more heavily in the parieto-occipital (hMT+, visual cortices, V1/V2/V4) regions involved in task performance. Note that a value of *H* was computed per cortical label here. See La Rocca et al. (2018b) for methodological details.

In La Rocca et al. (2018b), selfsimilarity was systematically quantified by wavelet-based measurements of the selfsimilarity exponent *H* and a global decrease from rest to task was observed over the whole brain (see Figure 4E in La Rocca et al., 2018b). This result, obtained from 24 participants, is here strengthened by using 36 subjects. Figure 9 reports a decrease in *H* not only between REST_*i*_ and TASK_*i*_ but also between REST_*i*_ and TASK_*f*_. Further, Figure 9 shows a strengthening of the decrease in *H* from TASK_*i*_ to TASK_*f*_ in the parieto-occipital regions involved in task performance, notably the bilateral hMT+ regions, the visual cortices including V1/V1 and V4 for the visual color detection. Interestingly, after training, these regions are also more strongly coupled with others during task performance (TASK_*f*_ vs. REST_*i*_).

**Figure 10:**
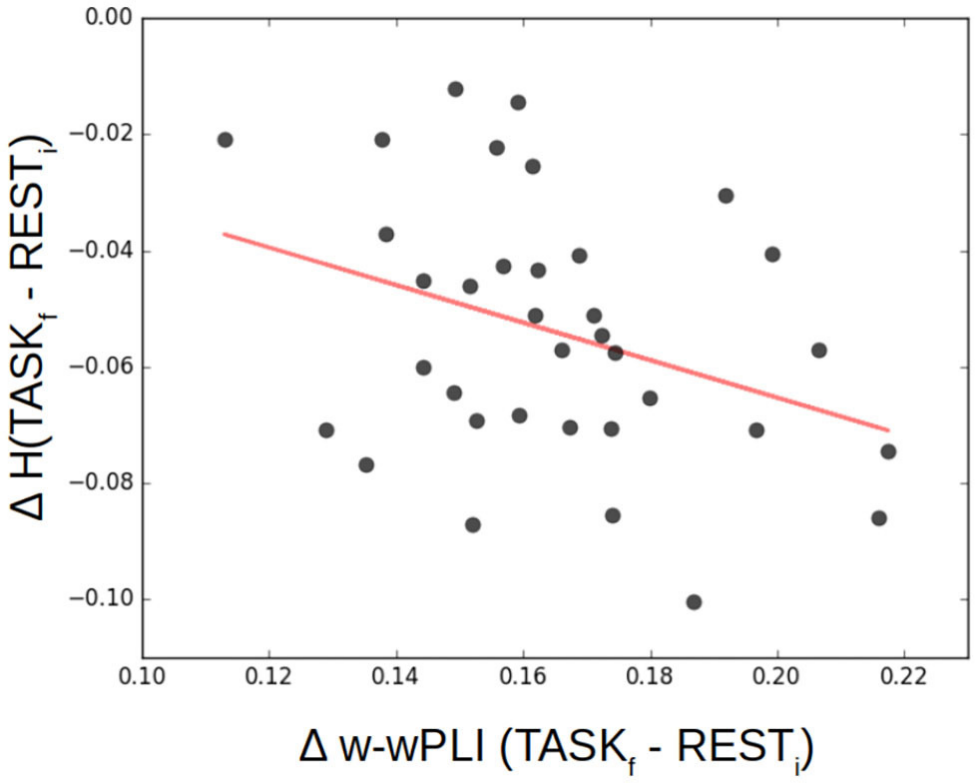
Decrease of selfsimilarity vs. increase in functional connectivity assessed from fractal dynamics from rest to task. Δ*H*= H_TASK_*f*__-H_REST_*i*__ as a function of Δ W-wPLI = W-wPLI_TASK_*f*__ − W-wPLI_REST_*i*__, averaged across the whole brain for each of the 36 participants (each marked as a dot), shows that the decrease of selfsimilarity correlates negatively (*r* = −0.33, *p* = 0.05) with the increase of functional connectivity assessed from fractal dynamics.

To investigate a potential training-induced relation between the decrease in selfsimilarity and the increase in W-wPLI, Δ*H*= H_TASF_*f*__ − H_REST_*i*__ and ΔW-wPLI = W-wPLI_TASF_*f*__ − W-wPLI_REST_*i*__ were averaged across the whole brain for each subject. Corresponding averages are shown in Figure 10 which interestingly suggests a significant (*p* = 0.05) anticorrelation of *r* = −0.33. When averages are restricted to the part of the brain where statistically significant changes in W-wPLI between REST_*i*_ and TASK_*f*_ can be assessed (after false discovery rate-based corrections for multiple hypothesis testing), the relation between Δ*H* and Δ W-wPLI is strengthened, *r* = −0.35 and *p* = 0.04.

### 4.4. Functional Connectivity Assessed From Fractal Dynamics and Task Performance

**Figure 11:**
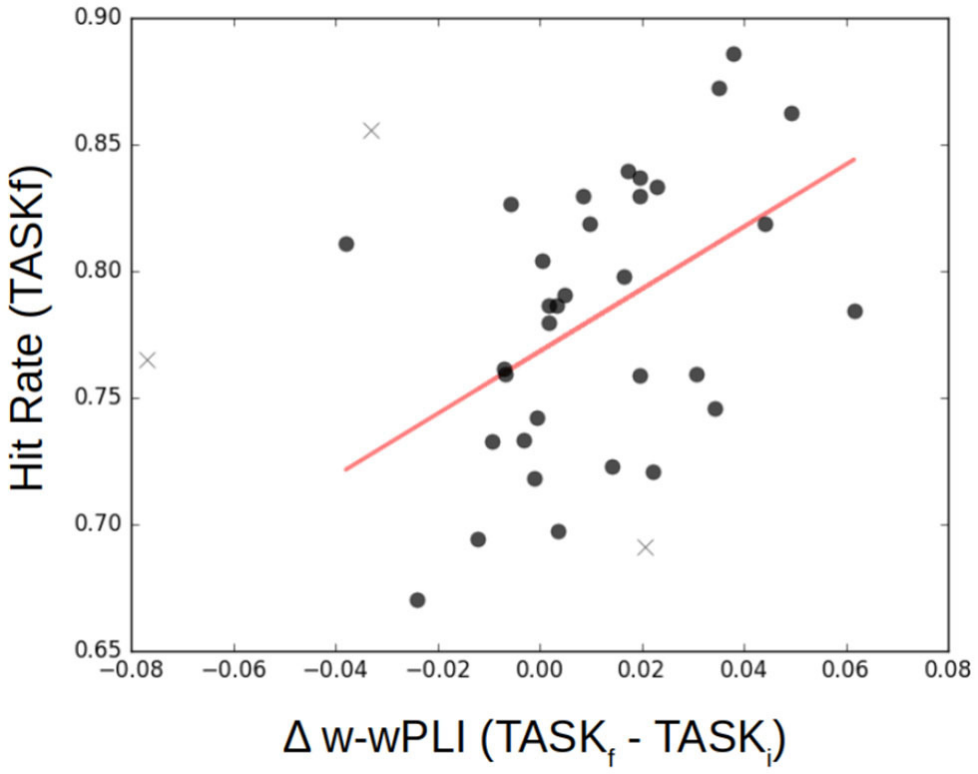
Functional connectivity assessment from fractal dynamics vs. Task Performance. Individual performance in the post-training task shows significant (*p* = 0.01) positive correlation (*r* = 0.45) with the difference in functional connectivity assessed from fractal dynamics from pre- to post-training, i.e., W-wPLI_TASK_*f*__ − W-wPLI_TASK_*i*__. Each participant is represented as a dot, and outliers are marked with a ×.

Finally, functional connectivity in the infraslow range of temporal dynamics can be related to task performance, and this is notable after training. Figure 11 reports, for each participant, post-training performance in achieving the task quantified by a percentage of correct responses (detection of the color associated with the coherent visual motion), referred to as hit rate, as a function of the variation in the W-wPLI indices measured in TASK_*i*_ and TASK_*f*_. It shows that participants with the larger increase in functional connectivity assessed from fractal dynamics induced by training, i.e., the larger increase of W-wPLI_TASK_*f*__ − W-wPLI_TASK_*i*__, are also those achieving the better performance in post-training task.

### 4.5. Functional Connectivity From Fractal Dynamics: Fourier-Based vs. Complex-Wavelet Assessment

**Figure 12:**
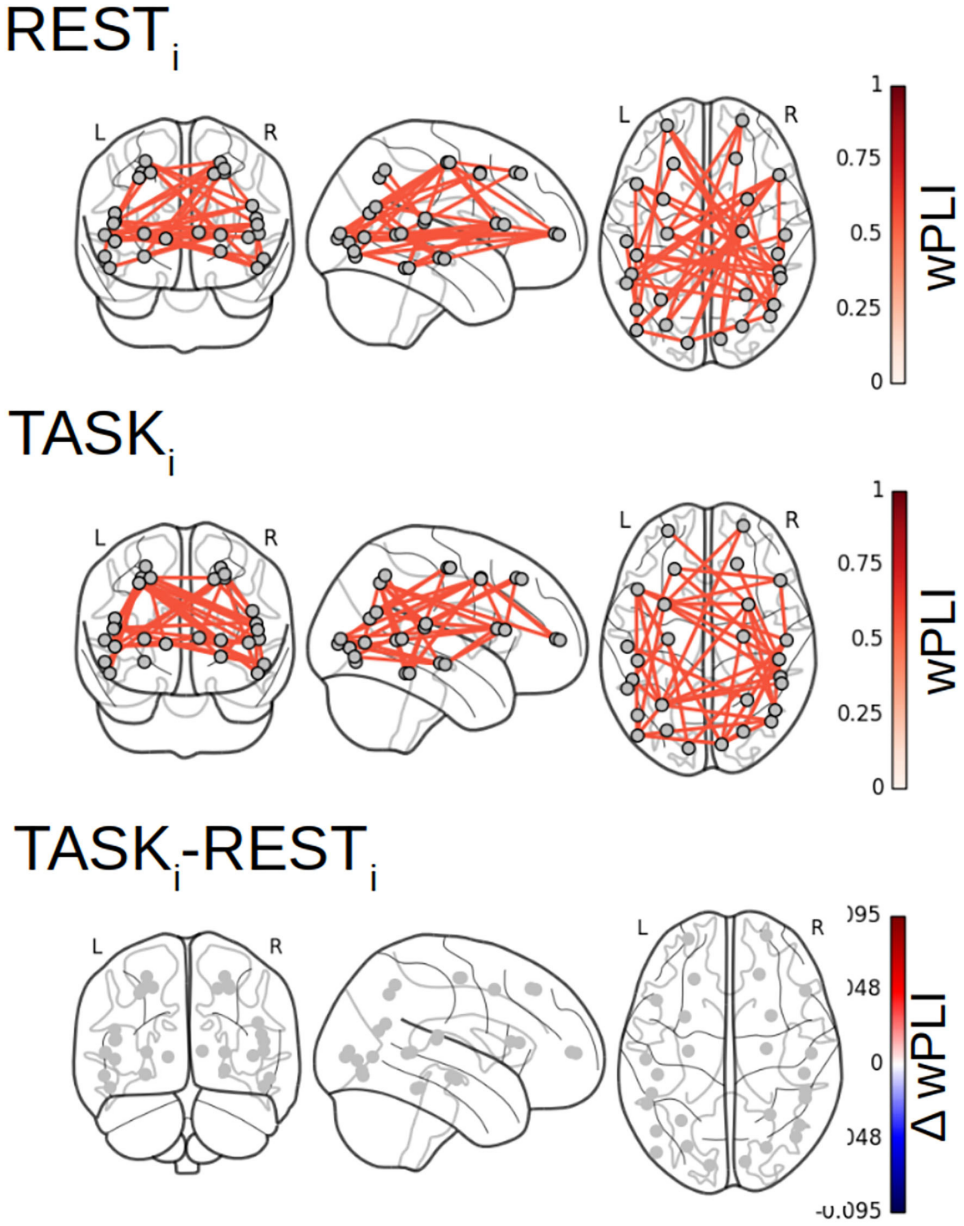
Fourier-based wPLI estimator in the scale-free regime. No significant difference between F-wPLI_TASK_*i*__ and F-wPLI_REST_*i*__ in arrhythmic regime can be found.

Averaging (the absolute values) of F-wPLI across a range of frequencies that match the range of scales associated with the infraslow scale-free scaling range permits us to compare Fourier-assessed functional connectivity from fractal dynamics. Figure 12 reports the density networks obtained from F-wPLI for REST_*i*_ and TASK_*i*_, showing significant differences with those obtained using W-wPLI. The network topography associated with the F-wPLI index are denser compared to W-wPLI. Indeed, using the Average Degree, used as a graph structure metric, it was found that for REST_*i*_, the Average Degrees of W-wPLI and F-wPLI are 0.44(±0.52) and 1.62(±1.11), respectively, yielding a very significant difference, assessed by a *p*-value below 6 × 10^−6^, and for TASK_*i*_, the Average Degrees of W-wPLI and F-wPLI are 0.52(±0.50) and 1.65(±1.21), respectively, yielding also a significant difference assessed by a *p*-value of 5 × 10^−5^. Further, the number of significant interactions with F-wPLI is more balanced between the two hemispheres during REST_*i*_ in contrast to W-wPLI, which captures more couplings in the right one. Also, the resting-state W-wPLI-based network configuration is more dominated by fronto-occipital couplings, whereas the F-wPLI-based shows a greater number of inter-hemispheric interactions. During the pre-training task TASK_*i*_, the W-wPLI and F-wPLI network topographies both show similar connections but also strong differences: the former is more dominated by fronto-parieto-occipital couplings with a hub role played by the visual cortices, while the latter does not strongly differ from the F-wPLI network found during REST_*i*_. Finally and more importantly, no statistically significant difference in F-wPLI_TASK_*i*__-F-wPLI_REST_*i*__ can be evidenced (see Figure 12-bottom), while a significant increase in W-wPLI was found from REST_*i*_ to TASK_*i*_ between fronto-parieto-occipital regions that are involved in task performance (see Figure 9-top). The coupling between V4 and MT in the right hemisphere reflects the color-motion binding, while the significant interactions involving the anterior STS, IPS, and vlPFC are likely due to their role in multisensory processing. The W-wPLI index thus provides much more meaningful information when contrasting rest to task brain activity.

## 5. Discussion

### 5.1. Functional Connectivity From Fractal Dynamics Assessment

At the methodological level, the results presented in section 4 clearly showed that W-COH fails to characterized correctly functional connectivity, which is in clear agreement with the numerical simulations reported in section 2.3 on synthetic data fGn/fBm and with results reported in the literature (cf. Stam et al., 2007; Vinck et al., 2011).

More interestingly, compared to W-ICOH, W-wPLI was observed to more accurately quantify functional connectivity assessment from fractal dynamics, both at rest and during a task in MEG data, as well as to better highlight relevant changes in functional connectivity assessed from fractal dynamics between rest and task. This is in agreement with previously reported results, showing that for band-limited oscillatory activities, F-wPLI was a better index to assess functional connectivity than F-ICOH. This was attributed to the denominator of F-wPLI being different from that of F-ICOH and less sensitive to (residual) volume conduction effects (Stam et al., 2007; Vinck et al., 2011). These arguments straightforwardly extend to W-wPLI and W-ICOH, and they thus likely explain the enhanced ability of W-wPLI to assess functional connectivity from fractal dynamics compared to W-ICOH. Interestingly, the numerical simulations conducted in section 2.3 on synthetic fGn/fBm data showed only a moderate superiority of W-wPLI over W-ICOH to quantify functional connectivity from fractal dynamics, except for slightly improved estimation (RMSE) performance. This suggests that fGn/fBm, even with delays, correlations, and possible additive trends, are not rich enough models to account for all the difficulties encountered in modeling real MEG data. This is calling for richer modeling, potentially involving multifractality. This will be further explored.

The benefits of using wavelet-based (multiscale) tools to analyze scale-free temporal dynamics and estimate the corresponding scaling exponent compared to classical Fourier-based spectral estimation have been abundantly documented elsewhere (cf. e.g., Abry and Veitch, 1998; Veitch and Abry, 1999, 2001; Ciuciu et al., 2008, 2012; Abry et al., 2019b). First, they provide better (unbiased and controlled variance) estimates of *H*; second, by tuning the so-called number of vanishing moments of the mother wavelet (Mallat, 1998), wavelet-based spectral estimation is robust to additive smooth slow trends in data which are, to the converse, strongly altering Fourier-based spectral estimation. These benefits are straightforwardly inherited by the wavelet-based indices for assessing functional connectivity from fractal dynamics. This was evidenced by the numerical simulations reported in section 2.3 showing the robustness of trends and improved performance for large scaling exponents of Complex Wavelet-based indices over Fourier-based ones.

### 5.2. Functional Connectivity Assessed From Fractal Dynamics in Time Relates to Long-Range Spatial Interactions

On MEG data, functional connectivity in the infraslow arrhythmic regime assessed by W-COH, i.e., based on direct correlation, was observed to yield mostly spatial short-range connectivity networks across the brain, notably with spurious short-range functional intra- and inter-hemispheric interactions, visible between frontal regions both at rest and during a task. This is likely a consequence of residual common source effects, strongly biasing the real part of thecoherence function, and thus yielding spurious connectivity measures, in agreement with results reported in Stam et al. (2007). In contrast, functional connectivity assessed by W-ICOH and W-wPLI indices, i.e., based on phase coupling, did not show such short-range links, but rather functional connectivity patterns dominated by long-range spatial interactions. This yields the first major result of the present work: Functional connectivity pertaining to the large-band infraslow arrhythmic temporal dynamics (from 1 to 10 s, or equivalently from 0.1 to 1 Hz), reveals long-range spatial interactions, notably evidencing couplings between frontal, parietal, and occipital brain regions. Functional connectivity assessed from fractal dynamics thus permits to quantify phase couplings and interactions associated with large lags. This departs from functional connectivity networks produced by the analysis of band-limited oscillatory temporal dynamics, that pertains to the fast (high frequency) brain activity and thus focuses on short time delays.

### 5.3. Functional Connectivity Assessed From Fractal Dynamics Increases During Task Performance and With Training

Compared to F-wPLI, W-wPLI showed an enhanced statistical sensitivity as it revealed a positively engaged parieto-temporo-occipital network in infraslow temporal dynamics when contrasting rest to pre-training activities. This network comprises previously identified key brain regions (e.g., hMT+, ITC, vlPFC, and pSTS) during task performance. Interestingly, such regions also consistently identified as beubg recruited by a task when using standard temporal or spectral data analysis (Zilber et al., 2014; La Rocca et al., 2020). However, W-wPLI was the only index further showing that functional connectivity assessed from fractal dynamics actually increased during task performance in these regions. A second key result consists of the observation of the strengthening of this functional connectivity from fractal dynamics based functional network with training, i.e., when contrasting rest to post-training activity. It shows the rising of new key couplings between frontal and parieto-temporal cortices, which suggests that some cortical representations of the visual detection and decision-making process may emerge even at slow time scales (1–10 s) and may be used as a substrate for facilitating faster dynamics in oscillatory regimes. Such increased functional connectivity assessed from fractal dynamics is a hallmark of brain plasticity induced by the training stage.

The third finding of this study is the positive correlation between the increase in functional connectivity assessed from fractal dynamics and task performance when contrasting pre- to post-training brain activity. This suggests that the consolidated network eases task completion for each individual, experiencing averaged increase in functional couplings within the infraslow regime.

### 5.4. Functional Connectivity From Fractal Dynamics and Selfsimilarity Quantifying an Interplay Between Temporal and Spatial Dynamics

Finally, the increase in functional connectivity assessed from fractal dynamics was shown to be correlated with a decrease in the selfsimilarity from rest to task. These results on functional connectivity assessment from fractal dynamics, combined with the univariate (regionwise) analysis of scale-free temporal dynamics of the same data (La Rocca et al., 2018b), lead to the following global picture for the large-band arrhythmic infraslow temporal dynamics of brain activity.

At rest, each region displays a globally very structured and slow activity in time (large selfsimilarity exponent *H* and thus strong temporal autocorrelation) with no transient structures (no burstiness and no multifractality, La Rocca et al., 2018b). The regions are connected across the brain by a clear spatial structure, that of functional connectivity assessed from fractal dynamics, constructed on measures of infraslow arrhythmic interactions.

During task performance, temporal dynamics in each region independently become less globally structured and faster (decrease in *H* hence globally less correlated) with transient dynamical structures for regions involved in the task (burstiness and multifractality, La Rocca et al., 2018b). These changes in regionwise temporal dynamics are accompanied by stronger functional connectivity assessed from fractal dynamics, i.e., by stronger spatial structures connecting regions.

This permits us to conjecture an interplay between temporal and spatial dynamics for the large-band infraslow arrhythmic brain activity: A decrease in global temporal structures induces faster and transient temporal dynamics and is associated with an increase in spatial structures and interactions between remote brain regions. Interestingly, these modulations are further strengthened with training, i.e., when contrasting the post-training to the resting-state activity in comparison with the pre-training vs. rest contrast. Overall, such modulations of brain spatio-temporal dynamics can be conjectured as a hallmark of brain plasticity.

## 6. Conclusions

In this work, we have introduced the notion of *functional connectivity assessment from fractal dynamics* for MEG data, defined as functional connectivity associated with the large-band infraslow (typically below the Hz) arrhythmic (scale-free) cross-temporal dynamics, in contradistinction with the classical functional connectivities associated with the band-limited rapid oscillatory rhythms (α−, β−, γ− bands).

It has been argued and demonstrated that complex wavelet (multiscale) based analyses permit to construct indices to assess functional connectivity from fractal dynamics that inherit from the theoretical and practical benefits of wavelet representations for scale-free (cross-temporal) dynamics analysis, notably in terms of robustness to trends and large selfsimilarity parameters *H*. It was confirmed that wPLI outperforms ICOH, as commonly observed, and that COH is not suited for functional connectivity assessment.

While Fourier-based tools are natural to use to assess functional connectivity in band-limited rapid oscillatory rhythms, it was shown, using simulated synthetic data and mostly on MEG data, that the assessment of functional connectivity for large-band slow scale-free cross-temporal dynamics is better achieved by complex wavelet based indices. Therefore, Fourier and complex wavelet-based spectral estimation must be regarded as complementary, rather than as mutually exclusive, tools.

Complex wavelet-based analyses of functional connectivity assessment from fractal dynamics conducted on MEG data recorded on 36 participants at rest and during a visual discrimination task with individualized training, yielded several key conclusions. First, large-band infraslow arrhythmic cross-temporal dynamics can be associated with long-range (fronto-temporo-occipital) spatial interactions. Second, functional connectivity from fractal dynamics increases during task performance (in a set of brain regions consistent with those evidenced by other analyses performed on the same data with different tools) and is strengthened with training. Interestingly, a larger overall fractal dynamics-based functional connectivity increase correlates with better task performance (larger hit rate). Third, the increase in spatial structure (quantified by the increase in functional connectivity assessed from fractal dynamics) is accompanied by changes in temporal structures, combining a decrease in the global temporal correlations (quantified by a decrease in the selfsimilarity index) and the increased occurrence of local transient structures (quantified by an increase in multifractality). These spatiotemporal modulations are reinforced with intensive and individualized training for the task.

Routines (in Matlab) to synthesize (correlated and delayed) bivariate fractional Gaussian noise, to perform Fourier and complex-wavelet based analysis and to compute indices quantifying functional connectivity from fractal dynamics, on synthetic or MEG data, have been developed by ourselves and will be made publicly available at the time of publication.

Such tools could further be used to examine the relevance of functional connectivity assessed from fractal dynamics in the context of network physiology, and networks of networks, relating brain activity to other physiological functions (heart rate, respiration, sleep, ocular, and motor systems, etc.) (cf. e.g., Bartsch and Ivanov, 2014; Bartsch et al., 2015; Liu et al., 2015; Catrambone et al., 2020).

## Data Availability Statement

The data analyzed in this study is subject to the following licenses/restrictions: neurospin property. Requests to access these datasets should be directed to patrice.abry@ens-lyon.fr.

## Ethics Statement

The studies involving human participants were reviewed and approved by Local Ethics Committee on Human Research at NeuroSpin (Gif-sur-Yvette, France) Protocole CPP_100022_Cognitif. The patients/participants provided their written informed consent to participate in this study.

## Author Contributions

The original experimental design and access to MEG data was provided by VW. The methodological question studied here was framed and conceptualized by PA and PC. The data analysis tool design, implementation and performance assessment, and interpretation were performed by HW and PA. MEG data analysis, results production, and interpretation were performed by DLR and PC. The article was written by PA and PC. All authors contributed to the article and approved the submitted version.

## Conflict of Interest

The authors declare that the research was conducted in the absence of any commercial or financial relationships that could be construed as a potential conflict of interest.
